# Different Incidence of Early-Onset Gastric Carcinoma Depending on Ethnicity: Preliminary Results of a Hospital in Liangshan

**DOI:** 10.1155/2020/6845413

**Published:** 2020-03-10

**Authors:** Shen Li, Peter Rexin, Zhang Qin, Chen Changbo, Chen Guanghui, Wang Luyao, Hans-Ullrich Voelker, Gerhard Stauch

**Affiliations:** ^1^Department of Pathology, No. 1 Hospital Liangshan, Xichang, China; ^2^Department of Pathology Aurich Westerstede, Westerstede, Germany; ^3^Department of Gastroenterology, No. 1 Hospital Liangshan, Xichang, China; ^4^Department of Foreign Affairs, No. 1 Hospital Liangshan, Xichang, China; ^5^Pathology, Leopoldina Hospital Schweinfurt, Schweinfurt, Germany; ^6^Senior Expert Service Bonn, Bonn, Germany

## Abstract

**Background:**

In China, the incidence of cancer has significantly decreased over the last two decades. In contrast, the incidence of gastric carcinoma (GC) has risen in young patients.

**Methods:**

We reevaluated the histopathological results of 4,353 endoscopic gastroscopies from the Department of Pathology at No 1 Hospital of Liangshan. The ethnic groups Han and Yi were almost equally distributed in this cohort. Over a five-year period, 1407 GC were diagnosed.

**Results:**

In 171 of these cases (12%), the patients were ≤40 years old (early-onset GC, EOGC). Out of this cohort, 9 patients were aged ≤25 years. 54% of these patients were male and showed marked predominance (92%) of the Yi-minority. Using the classification of Lauren, 103 GC (60%) were of diffuse type, 27 (16%) of intestinal type, and 41 (24%) of mixed type. In the remaining 1,236 cases of patients ≥41 years (88%), 1,014 patients (82%) belonged to the Yi-minority. *Helicobacter pylori* (HP) were found in 46% of all cases. Familial clustering was found in 14 patients (18%; in first degree relatives, 12%, and in second degree relatives, 6%). Follow-up was not possible.

**Conclusion:**

This study demonstrates the unequal manifestation of EOGC within the two ethnic groups of Han and Yi. However, familial clustering was infrequent. Further investigations are necessary to discover relevant risk factors apart from hereditary predisposition.

## 1. Introduction

Gastric carcinoma (GC) is the fourth most common type of cancer with a high rate of cancer-related deaths despite manifold therapeutical efforts [1]. In China, its incidence has significantly decreased over the last two decades, mostly in urban and to some extent in rural areas. However, an increasing number of cases can be found in young patients under 40 years [2, 3], which is classified as early-onset GC (EOGC).

Over the last five decades, the histological classification of GC has been largely based on Lauren's criteria [4] with discrimination of the intestinal-, diffuse-, and indeterminate mixed-type. The literature describes the incidences of approximately 50% for the intestinal-, 35% for the diffuse-, and 15% for the mixed-type. Lauren's classification is currently accepted worldwide as a simple and robust approach for the determination of histological subtypes, which exhibit a number of distinct clinical characteristics including epidemiology, aetiology, tumorigenesis, cell differentiation, biological behavior, and prognosis [5–7].

GC research is hampered by the diversity of factors which can induce tumor growth. These include numerous exogenous and environmental factors such as bacterial infections with *Helicobacter pylori* (HP), variations of lifestyle, and diet [8–14] as well as genetic or epigenetic abnormalities which affect tumor suppressor genes and mismatch repair genes [15]. In this way, clinical research is faced with a seemingly unsolvable puzzle. It is generally assumed that GC in young patients is more often induced by genetic and epigenetic alterations, in contrast to the predominance of environmental factors in older patients.

In 2017, the author GS noticed in own unpublished observations a high incidence of EOGC within the histologically verified cases of GC in the Department of Pathology of No 1 Hospital of Liangshan in Xichang. Based on this impression, we carried out a pilot study to examine the distribution of patients with regard to the ethnic groups Han and Yi. Furthermore, we investigated the degree of infection by HP and the familial risk of the affected patients.

## 2. Materials and Methods

Between April 2013 and April 2018, the Department of Pathology of No 1 Hospital investigated the biopsy specimens from 4,353 endoscopic gastroscopies histologically. In this cohort, the distribution to ethnicity of patients to Han and Yi was analyzed; this assignment to ethnic groups was also made for cases of GC. All patients were residents of Liangshan prefecture. New slides of every block of paraffin-embedded tumor specimens were manufactured and stained with hematoxylin-eosin (H&E), Giemsa, and periodic acid-Schiff-diastase (PAS-D). The gastric carcinomas were histologically reevaluated and classified in terms of Lauren and the recent WHO classification by the authors SL and GS in June/July 2018.

The patients in the EOGC group of ≤40 years were subgrouped as follows: ≤20, 21–25, 26–30, 31–35, and 36–40 years. The patients in the GC group of ≥41 years were subgrouped as follows: 41–50, 51–60, and ≥61 years.

The rate of HP infection for each group and tumor type was evaluated histologically using the Giemsa stain by two experienced pathologists.

In the 77 patients of the EOGC group ≤40 years, the documentation of an individual and familial case history was possible by phone interview and by tracing hospital documents.

## 3. Results

**Figure 1:**
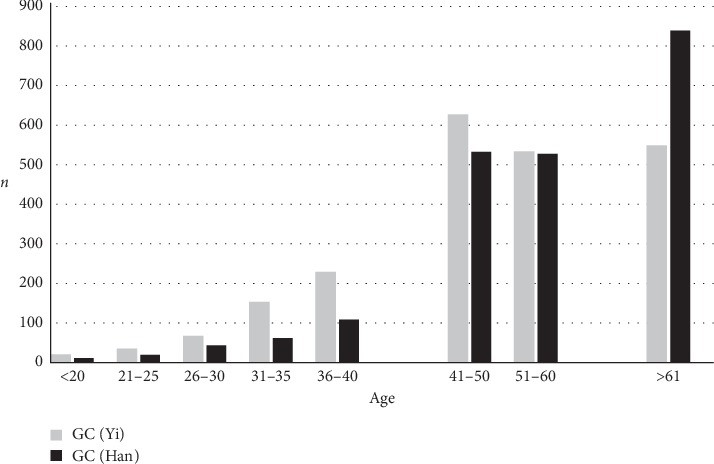
Number of endoscopic evaluation of stomach by taking biopsies for histological investigations in patients of Yi and Han ethnicity.

The distribution of ethnic groups Han and Yi, the ages of patients, and the histological results of endoscopy from 4353 cases are shown in Figure 1. 2143 (49.2%) biopsy specimens were from the Han-majority and 2210 specimens (50.8%) from the Yi-minority, so that a sampling bias could be excluded.

In 1407 cases (32.3%), the biopsy specimens from gastroscopy contained GC. 171 of these tumor specimens (12%) originated from patients ≤40 years, so that an EOGC was diagnosed.  Table 1 shows the distribution of age, tumor type, and ethnic group. The prevalence of the Yi-population in EOGC was 94% and in the cohort of patients ≥41 years was 88% (1088 cases). The most striking result was that 9 patients from 14–25 years with EOGC showed 100% Yi ethnicity.

In 77 of 171 patients with EOGC (44%), it was possible to analyze the oncologic history of close relatives. 14 patients of Yi-minority showed tumors in 9 first-degree relatives and 5 second-degree relatives. All tumors of these 14 patients were of diffuse- or mixed-type according to Lauren (Table 2). The examination of exogenous and environmental factors, as far as possible in anamnesis, showed no difference in the quantity of alcohol and nicotine consumption between Han- and Yi-population. However, the traditional diet differs between Han and Yi with the latter preferring more salted smoked meat and fermented vegetables.

## 4. Discussion

The available information about gastric carcinoma (GC) is complicated by a host of partially interdependent aetiological factors of exogenous and genetic nature. Exogenous factors can comprise bacterial, environmental, and dietary factors as well as lifestyle and exposure to toxic substances. They differ in the potency of malignant transformation. Exogenous factors are modulated by the immunological and genetic background of the host and differing genetic penetration, which may cause gene activation or suppression. In addition to sporadic carcinomas, there are 3–5% purely hereditary carcinomas defined by autosomal dominant mutations. The frequency of tumor patients of <25 years in this group strongly supports this theory. Guiford et al. already revealed in 1986, the genetic background in young patients [11, 12]. A familial clustering such as in the Yi-families was already found in studies of some Maori-families in New Zealand. Other results of different study groups revealed a genetic GC disposition in juvenile patients with Li–Fraumeni syndrome, Lynch syndrome, Peutz–Jeghers syndrome, juvenile polyposis syndrome, or Cowden syndrome [14].

The Yi-population is a minority of more than 6 million people, mostly living in Sichuan and Yunnan with a relatively homogeneous genetic background, mostly because of the traditional separation from the surrounding Han-majority by social rules, different language, different religion, and their remote living situation in mountainous and inaccessible areas. Familial clustering may be a strong sign of genetic influence, but additional exogenous factors such as hygiene and traditional food habits with consumption of salted and smoked red meat and fermented fish and pickles cannot be excluded [16, 17]. With increasing assimilation between ethnicities, the influence of genetic similarity will decrease.

The most important exogenous factor in GC induction is an infection with *Helicobacter pylori* (HP), which is controlled by the oncogenicity of HP CagA, Epuiya-c, and msi VacA types as well as the genetic susceptibility of hosts [18]. This is mainly true for sporadic carcinoma. Carcinoma can develop in the setting of long-term exposure to HP via a cascade of morphological changes. In cases of EOGC, tumor induction has, however, also been associated with a high incidence of HP. In almost all studies, up to 80% of GC patients are infected with HP (see Table 3) [16, 17, 19]. In our study, HP was found in less than 46%. These results may be blurred by preferential sampling of specimens directly from the neoplastic area. Biochemical control measurements were not performed in this study. Especially, newer studies of EOGC have shown an increased incidence of HP infection, which suggests this as a cofactor for malignant transformation. To what extent early and consistent eradication of HP in children can contribute to minimizing GC incidence in these risk groups must be evaluated by further investigations.

A stage classification of the GC was not possible in our cohort as comprehensive data concerning pretherapeutic staging (computer tomography) were not available, and, respectively, only 33 patients (19%) underwent further therapeutical procedures. Both overall survival and disease-free survival could not be determined in this study. Detection methods of CDH1 germline mutations were also not feasible in the time frame of the study.

The high percentage of patients of the Yi-minority both in the EOGC group and in the GC group <40 years has not been described up to now. However, individual families with gastric carcinoma clustering were identified by Guiford et al. in New Zealand, as well as by other groups in Italy. Only Bai and Li have described a significant tumor clustering in a Chinese study [19]. Susceptibility of individual ethnic groups seems to play a decisive role for the oncogenic potency of some HP strains, in particular the Cag and msi VacA groups, in which malignant transformation into intestinal carcinoma types is frequently expected [15].

In the cohort presented here, the authors could not find significant differences in lifestyle and alcohol or nicotine consumption in contrast to Lee et al. [18]. However, we observed different eating habits in the Yi-population with a preference for smoked and salted meat and fermented vegetables whose carcinogenicity was particularly highlighted by Japanese study groups [20]. The dietary differences are, however, of increasingly minor importance because the Yi have been assimilated more and more to the Han lifestyle in the last decades.

## 5. Conclusion

Our study gives a first insight into differences in the development of early-onset gastric cancer in different ethnic groups with a distinctly higher incidence of this disease in the Yi-population. Apart from lifestyle parameters, diet, and frequency of *Helicobacter pylori* infections, further studies are necessary to reveal the genetic background of this phenomenon with the aim to identify families or individuals with a higher risk for gastric cancer, so that these could be included in programs for early detection.

## Figures and Tables

**Table 1:** Early-onset gastric carcinomas in the 1407 reevaluated samples of all included gastric carcinomas.

Age	*n*	Gender		Lauren type			Yi-minority
		Male	Female	Diffuse	Intestinal	Mixed	
≤20	4	3 (75%)	1 (25%)	4 (100%)	—	—	4 (100%)
21–25	5	4 (80%)	1 (20%)	4 (80%)	—	1 (20%)	5 (100%)
26–30	21	14 (67%)	7 (33%)	14 (67%)	4 (19%)	3 (14%)	19 (90%)
31–35	43	26 (60%)	17 (40%)	29 (67%)	6 (14%)	8 (19%)	42 (98%)
36–40	98	53 (54%)	45 (46%)	52 (53%)	17 (17%)	29 (30%)	88 (90%)
∑	171	100 (59%)	71 (41%)	103 (60%)	27 (16%)	41 (24%)	158 (92%)

**Table 2:** Gastric carcinoma (GC) in grade 1 and grade 2 relatives of 77 interviewed patients.

Gender	Age	Cancer type	Relative grade 1	Relative grade 2
M	24	D	GC Mo	
F	25	D	GC Fa + GC Si	
F	26	D		GC Un
F	40	Mi		Tumor not classifiable
F	38	D	GC Sib	
M	38	D	GC Sib	
F	37	Mi		GC Un
F	38	D		GC 3X Un
F	40	D	GC Mo	
M	40	D	GC Mo	
F	37	D	GC Sib + GC Fa	
M	39	Mi		GC Un
M	40	Mi	GC Sib	
M	39	D	GC Fa	

M-male, F-female, D-diffuse type of Lauren, Mi-mixed type of Lauren, Fa-father, Mo-mother, Si-sister, and Un-uncle.

**Table 3:** Literature review from three studies compared with the own study.

Author	Bay et al. [19], China	Kono et al. [16], Japan	Quach et al. [17], Vietnam	Own study
Study design	Prospective	Retrospective	Prospective	Retrospective
Number of cases	250	72	141	171
Age median	31	35	35	37
Gender male/female ratio	0.66	1.1	1.109	1.1
Diffuse type of Lauren	64%	92%	87%	88%
*Helicobacter pylori*	Not reported	81%	73%	46%
Clinical stage	Not reported	III-IV	III-IV	Not reported
Familial history	19%	10%	0	12%

## Data Availability

All data are given in the study. Material is archived following the rules of Hospital No 1, Liangshan.

## References

[1] Sitarz R., Skierucha M., Mielko J., Offerhaus J., Maciejewski R., Polkowski W. (2018). Gastric cancer: epidemiology, prevention, classification, and treatment. *Cancer Management and Research*.

[2] Yin J., Song J. N., Bai Z. G. (2017). Gastric cancer mortality trends in China 2006–2013 reveal an increasing mortality in young subjects. *Anticancer Research*.

[3] Zhu X., Li J. (2010). Gastric carcinoma in China: current state and future perspectives. *Oncology Letters*.

[4] Laurén P. (1965). The two histological main types of gastric carcinoma: diffuse and so-called intestinal-type carcinoma. *Acta Pathologica Microbiologica Scandinavica*.

[5] Qiu M.-Z., Cai M.-Y., Zhang D.-S. (2013). Clinicopathological characteristics and prognostic analysis of Lauren classification in gastric adenocarcinoma in China. *Journal of Translational Medicine*.

[6] Chen Y.-C., Fang W.-L., Wang R.-F. (2016). Clinicopathological variation of lauren classification in gastric cancer. *Pathology & Oncology Research*.

[7] Hu B., El Hajj N., Sittler S., Lammert N., Barnes R., Meloni-Ehrig A. (2012). Gastric cancer: classification, histology and application of molecular pathology. *Journal of Gastrointestinal Oncology*.

[8] Simán J. H., Forsgren A., Berglund G., Florén C.-H. (2001). Tobacco smoking increases the risk for gastric adenocarcinoma among *Helicobacter pylori*-infected individuals. *Scandinavian Journal of Gastroenterology*.

[9] Parsonnet J., Vandersteen D., Goates J., Sibley R. K., Pritikin J., Chang Y. (1991). *Helicobacter pylori* infection in intestinal-and diffuse-type gastric adenocarcinomas. *JNCI Journal of the National Cancer Institute*.

[10] Brenner H., Arndt V., Bode G., Stegmaier C., Ziegler H., Stümer T. (2002). Risk of gastric cancer among smokers infected with *Helicobacter pylori*. *International Journal of Cancer*.

[11] Guiford P., Hopkins J., Harraway J. (1998). E-cadherin germ line mutation in familial gastric cancer. *Nature*.

[12] Guilford P., Humar B., Blair V. (2010). Hereditary diffuse gastric cancer: translation of CDH1 germline mutations into clinical practice. *Gastric Cancer*.

[13] Wolf E.-M., Geigl J. B., Svrcek M., Vieth M., Langner C. (2010). Hereditäres magenkarzinom. *Der Pathologe*.

[14] Van Der Post R. S., Vogelaar I. P., Carneiro F. (2015). Hereditary diffuse gastric cancer: updated clinical guidelines with an emphasis on germline CDH1 mutation carriers. *Journal of Medical Genetics*.

[15] Bacani J., Zwingerman R., Di Nicolo N. (2005). Tumor microsatellite instability in early onset gastric cancer. *The Journal of Molecular Diagnostics*.

[16] Kono Y., Kanzaki H., Tsuzuki T. (2019). A multicenter observational study on the clinicopathological features of gastric cancer in young patients. *Journal of Gastroenterology*.

[17] Quach D. T., Ha D. V., Hiyama T. (2018). The endoscopic and clinico pathological characteristics of early onset gastric cancer in Vietnamese patients. *Asian Pacific Journal of Cancer Prevention*.

[18] Lee S.-A., Kang D., Shim K., Choe J., Hong W., Choi H. (2003). Effect of diet and helicobacter pylori infection to the risk of early gastric cancer. *Journal of Epidemiology*.

[19] Bai Y., Li Z.-S. (2011). Endoscopic, clinicopathological features and prognosis of very young patients with gastric cancer. *Journal of Gastroenterology and Hepatology*.

[20] Shikata K., Kiyohara Y., Kubo M. (2006). A prospective study of dietary salt intake and gastric cancer incidence in a defined Japanese population: the Hisayama study. *International Journal of Cancer*.

